# The efficacy and clinical significance of microsurgery on cognitive function, activities of daily living, and serum biomarkers in patients with glioma

**DOI:** 10.12669/pjms.41.10.12018

**Published:** 2025-10

**Authors:** Yan Chen, Zhanhu Ma, Dan Zhao, Lei Mi, Xueshan Sun

**Affiliations:** 1 Yan Chen Department of Neurosurgery, Baoding No.1 Hospital, Baoding 071000, Hebei, China; 2 Zhanhu Ma Department of Neurosurgery, Baoding No.1 Hospital, Baoding 071000, Hebei, China; 3 Dan Zhao Department of Laboratory, Baoding No.1 Hospital, Baoding 071000, Hebei, China; 4 Lei Mi Department of Laboratory, Baoding No.1 Hospital, Baoding 071000, Hebei, China; 5 Xueshan Sun Department of Laboratory, Baoding No.1 Hospital, Baoding 071000, Hebei, China

**Keywords:** Microsurgery, Glioma, Cognitive function, ADL, Serum biomarker, Treatment

## Abstract

**Objective:**

To evaluate the efficacy of microsurgery on cognitive function, activities of daily living (ADL), and serum biomarkers in patients with glioma.

**Methodology:**

This was a retrospective study. A total of 120 eligible patients with glioma were enrolled from Baoding No.1 Hospital from May 2023 to December 2024 and randomly divided into two groups. The control group underwent conventional craniotomy for tumor resection, while the study group opted for microsurgery. The surgical outcomes were compared between the two groups. Additionally, levels of inflammatory markers (interleukin-6 [IL-6], serum amyloid A [SAA], C-reactive protein [CRP], and tumor necrosis factor-alpha [TNF-α]) and cytokines (arginine vasopressin [AVP], oxytocin [OT], and β-endorphin [β-EP]), cognitive function, and ADL were assessed before and after surgery.

**Results:**

The surgical outcomes of study group were significantly superior to control group. After treatment, levels of IL-6, CRP, SAA, and TNF-α were significantly lower in the study group than in the control group, while the levels of AVP, OT, and β-EP were significantly higher in the study group (*p* = 0.00, respectively). Postoperative cognitive function, including language, memory, and orientation, was significantly better in the study group than in the control group (*p* = 0.00). At three and six months postoperative, the ADL scores in the study group were higher than in the control group (*p* = 0.00).

**Conclusion:**

Microsurgery is an effective, efficient treatment modality for the treatment of glioma, which is worthy of clinical promotion.

## INTRODUCTION

Glioma is a common type of primary malignant intracranial tumor, accounting for approximately 40% of all intracranial tumors and 27% of central nervous system tumors.^1^ It is highly invasive and tends to spread from the primary site to normal brain tissue, making complete tumor resection challenging and severely affecting treatment outcomes.^2^ Glioma can lead to increased intracranial pressure, nausea, vomiting, headaches, seizures, and blurred vision, ultimately affecting neurological function and causing declines in cognitive function and activities of daily living (ADL).^3^ Traditional craniotomy with large bone flaps for tumor removal has several disadvantages, including large trauma, low visibility for the naked eye, and a high risk of damaging normal brain tissue.^4^ In recent years, traditional large-bone-flap craniotomy for tumor resection has gradually been replaced by microsurgical tumor removal under a microscope.

Microsurgery allows for more precise manipulation of tissues, accurate localization, and visualization of the tumor and its boundaries, leading to a higher rate of complete tumor resection.^5^ This improves surgical outcomes and extends the patient’s progression-free survival. Studies have shown that tumor development is closely related to inflammatory responses and cytokines, and the degree of change in these factors is associated with postoperative cognitive function and ADL in patients with glioma.^6^ On this basis, this comparative analysis of microsurgery and traditional open surgery in the treatment of glioma was conducted, and the findings suggest that microsurgery offers certain advantages, as reported below:

## METHODOLOGY

This was a retrospective study. A total of 120 patients with glioma who met the inclusion criteria were enrolled from Baoding No.1 Hospital May 2023 to December 2024 and randomly divided into the control group underwent conventional craniotomy for tumor resection and the study group opted for microsurgery (n=60 each group).

### Ethical Approval:

The study was approved by the Institutional Ethics Committee of Baoding No.1 Hospital (No.:2023-122; Date: April 20, 2023), and written informed consent was obtained from all participants.

### Inclusion criteria:

- Patients diagnosed with primary glioma according to the diagnostic standards.^7^
- First-time surgery for glioma treatment.
- Patients who agreed to participate in the study and signed informed consent forms.
- Tumors classified as grades I–IV according to the WHO glioma grading standards.^8^

### Exclusion criteria:

- patients with severe liver or kidney dysfunction or other organic diseases.
- patients with a history of cranial surgery.
- patients who were non-compliant with follow-up or had psychiatric disorders or cognitive impairments.
- pregnant or lactating women;
- patients with incomplete clinical data.

### Treatment methods:

Before surgery, both groups of patients underwent computed tomography or magnetic resonance imaging to determine the tumor location. Patients in the control group underwent conventional craniotomy for tumor resection. The patients were placed in the supine position. Under general anesthesia, the bone flap was removed, the dura mater was opened, and the tumor was resected. The bone flap was preserved or replaced based on the condition of brain tissue bulging after tumor removal.

Patients in the study group underwent tumor resection under the microscope. A conventional craniotomy was performed at a location near the tumor, with the dura mater opened. The surgery was performed via the coronal or pterional approach, avoiding major blood vessels and functional areas. After exploration, the tumor was accessed from the surrounding glial proliferation or edema region and resected from the center toward the periphery. After hemostasis and confirmation that no abnormalities were present, the incision was sutured, and the surgery was completed.

### Outcome measures:

### Surgery-related parameters:

Duration of operation (DoO), intraoperative blood loss (IBL), length of hospital stay (LoHS), incision length (IL), ambulation time (AT).

### Serum biomarker levels:

Peripheral venous blood was drawn from both groups of patients before and after surgery. Enzyme-linked immunosorbent assay (ELISA) was used to measure the levels of inflammatory markers (interleukin-6 [IL-6], serum amyloid A [SAA], C-reactive protein [CRP], and tumor necrosis factor-alpha [TNF-α]) and cytokines (arginine vasopressin [AVP], oxytocin [OT], and β-endorphin [β-EP]).

### Cognitive function:

Cognitive function was assessed using the Mini-Mental State Examination (MMSE)^9^ before surgery and three months after surgery for both groups. The MMSE includes three dimensions: language, memory, and orientation, with each dimension scoring from zero to five points. Higher scores indicate better cognitive function.

### ADL:

ADL was evaluated using the Instrumental Activities of Daily Living Scale (IADL)^10^ preoperative, three months postoperative, and six months postoperative for both groups. The total score of the IADL scale is 100 points. The higher the score, the better the ADL.

### Statistical analysis:

Statistical analysis was performed using SPSS 20.0. Measurement data were represented by (*χ̅*±*s*). Between-group comparisons were performed using an independent sample t-test. Within-group comparisons were conducted using a paired t-test. Repeated measurement data were analyzed using analysis of variance (ANOVA). Comparisons of rates were performed using the chi-square (*χ*²) test. A p-value of less than 0.05 was considered statistically significant.

## RESULTS

No significant differences were observed in the baseline data between the two groups, indicating a high level of comparability (Table-I).

**Table-I:** Comparative analysis of general patient data between the study and control groups (*χ̅*±*S*), n = 60.

Item	Study group	Control group	t/χ^2^	p-value
Age (years)	47.32±5.71	47.84±5.36	0.51	0.60
Male (n, %)	38 (63%)	36 (60%)	0.14	0.71
Disease duration (months)	5.26±1.33	5.42±1.27	0.67	0.50
Past history			0.05	0.82
Hypertension	13(22%)	12(20%)		
Diabetes	8(13%)	11(18%)		
Hyperlipidemia	14(23%)	15(25%)		
BMI (kg/m²)	23.48±3.15	23.73±3.09	0.44	0.67
Glioma grading			0.59	0.44
Grade I–II	41(68%)	37(62%)		
Grade III–IV	19(32%)	23(38%)		

p > 0.05.

The study group demonstrated significantly shorter DoO, less IBL, shorter LoHS, shorter average IL, and faster AT compared with the control group (*p* = 0.00, respectively) (Table-II). Before treatment, there were no statistically significant differences between the two groups in inflammatory markers (IL-6, CRP, SAA, TNF-α) (all *p* > 0.05). After treatment, these markers were significantly lower in the study group than in the control group (*p* = 0.00, respectively) (Table-III).

**Table-II:** Comparison of surgery-related parameters between the two groups (*χ̅*±*S*), n = 60.

Group	DoO (min)*	IBL (ml)	AT (d)*	IL (cm)*	Postoperative LoHS (d)*
Study group	65.83±9.44	75.60±20.33	3.59±1.07	5.26±1.13	6.40±1.54
Control group	84.57±8.25	103.25±21.80	6.76±1.75	8.86±1.27	9.35±1.48
*t*-value	11.57	7.08	11.80	8.40	11.44
*p*-value	0.00	0.00	0.00	0.00	0.00

* p < 0.05.

**Table-III:** Comparison of inflammatory marker and cytokine levels before and after treatment between the two groups (*χ̅*±*S*), n = 60.

Inspection item	Time point	Study group	Control group	t-value	p-value
IL-6 (ng/L)	Preoperative	15.75±6.43	15.46±6.18	0.25	0.80
	Postoperative*	6.93±3.12	9.07±3.75	3.40	0.00
CRP (mg/L)	Preoperative	97.35±20.31	96.84±19.50	0.14	0.89
	Postoperative*	57.62±9.55	62.68±9.11	3.01	0.00
TNF-α (ng/L)	Preoperative	47.63±13.52	46.87±12.80	0.32	0.75
	Postoperative*	23.78±7.55	28.29±8.15	4.32	0.00
SAA (mg/L)	Preoperative	134.07±22.42	132.84±23.06	0.40	0.69
	Postoperative*	53.73±13.76	63.62±12.09	4.18	0.00
AVP (ng/L)	Preoperative	23.37±5.60	23.48±6.07	0.14	0.92
	Postoperative*	12.85±4.29	8.05±4.11	6.26	0.00
OT (ng//L)	Preoperative	8.84±2.18	8.71±2.42	0.31	0.76
	Postoperative*	3.76±0.71	2.83±0.46	8.52	0.00
β-EP (ng/L)	Preoperative	96.59±10.44	97.23±9.87	0.34	0.73
	Postoperative*	63.18±8.72	54.06±8.02	5.96	0.00

* p < 0.05.

There were no significant differences between the two groups in AVP, OT, and β-EP levels before surgery (all *p* > 0.05). After surgery, these cytokine levels were significantly higher in the study group than in the control group (*p* = 0.00, respectively) (Table-III).

No significant differences were observed between the two groups in cognitive function, including language, memory, and orientation before surgery (all *p* > 0.05). At three months postoperative, the study group demonstrated significantly higher scores in these dimensions compared with the control group (*p* = 0.00, respectively) (Table-IV). No significant difference was observed in ADL scores between the two groups before surgery (all *p* > 0.05). At three and six months postoperative, the study group had significantly higher ADL scores than the control group (*p* = 0.00) (Table-V).

**Table-IV:** Comparison of cognitive function before and after surgery between the two groups (*χ̅*±*S*), n = 60.

Cognitive function	Time point	Study group	Control group	t-value	p-value
Language	Preoperative	1.68±0.14	1.67±0.13	0.41	0.69
	Postoperative*	3.74±0.70	3.21±0.53	4.68	0.00
Memory	Preoperative	1.73±0.15	1.71±0.21	0.60	0.55
	Postoperative*	3.58±0.62	3.19±0.57	3.59	0.00
Orientation	Preoperative	1.85±0.27	1.84±0.16	0.25	0.81
	Postoperative*	3.12±0.20	2.78±0.32	6.92	0.00

* p < 0.05.

**Table-V:** Comparison of ADL scores before and after surgery between the two groups (*χ̅*±*S*), n = 60.

Group	Preoperative	At 3 months postoperative*	At 6 months postoperative*	F-value	p-value
Study group*	60.75±5.63	70.53±6.19	78.27±6.82	9.05	0.00
Control group*	61.08±5.57	67.49±6.05	72.44±6.74	6.04	0.00
*t*	0.33	2.72	4.71		
*p*	0.74	0.00	0.00		

* p < 0.05.

## DISCUSSION

The results of this study demonstrate that microsurgery can effectively shorten surgery duration, hospital stay, and time to first postoperative ambulation, reduce intraoperative bleeding, and minimize incision size. These findings are consistent with previous studies.^11^ This effect may be attributed to the enhanced ability of microscopy to observe tumor characteristics, allowing for more accurate tumor localization. As the incision is smaller, patients also tend to experience faster postoperative recovery. A study reported the results of microsurgery on 20 cases of glioma, indicating that the final median resection rate was 92%, 60% of the patients were asymptomatic at discharge, and 45% of the patients were able to return to work after the operation.^12^ This also supports the conclusion of this study. OT, β-EP, and AVP are endogenous active substances found in neural tissues that can effectively promote the functional recovery of brain neurons.^13^ In this study, after surgery, the levels of OT, β-EP, and AVP in the study group were significantly higher than those in the control group. This suggests that microsurgery has a less damage to neural function. Sayyahmelli et al.^14^ also emphasized that microsurgery can provide safe and effective treatment for complex craniocerebral lesions. Inflammatory markers like IL-6, CRP, SAA, and TNF-α can induce M2 macrophage polarization, promoting glioma progression and playing an essential role in glioma development.^15^

Gliomas are primary intracranial tumors originating from glial cells, representing the most common type of primary brain tumor, accounting for approximately 80% of all malignant brain tumors.^16^ Its severely threaten patients’ lives, impact their quality of life, and impose heavy burdens on patients, their families, and society.^17^ Currently, surgery remains the mainstay of treatment for gliomas. However, craniotomy incisions are typically large, leading to excessive damage and resection of brain tissue in functional areas, which contributes to neurological dysfunction in patients. This affects postoperative outcomes, prolongs recovery time, leads to poor prognosis, and negatively impacts postoperative quality of life. Recently, microsurgery has gradually been applied to the treatment of patients with glioma. The microsurgical approach, which emphasizes precise operation, protection of blood vessels and nerves, preservation of normal tissue, and atraumatic suturing, is a key factor in surgical success.^18^

This study found that after the operation, the levels of the above inflammatory markers in the study group decreased more significantly than those in the control group. This confirms that microsurgery is safer than conventional open surgery and has a smaller impact on neural tissue. Studies suggested that circulating IL-6 and CRP may serve as powerful biomarkers for a poor prognosis in glioma patients.^19^,^20^ Further results showed that microsurgical treatment for glioma can improve cognitive function and ADL. This may be explained by the reduced damage to normal brain tissue during microsurgery, which allows for complete tumor resection with minimal impact on neural function, contributing to faster postoperative recovery for patients. This study systematically explored for the first time the combined effect of AVP, OT, β-EP and serum inflammatory factors (TNF-α, IL-6, CRP) in postoperative nerve injury and prognosis of patients with glioma, filling the research gap in this field.

### Limitations:

It include a small patient sample size and a short follow-up period. Future studies should increase the sample size and extend the follow-up duration to provide a more objective assessment of the long-term benefits and stronger clinical evidence of this treatment approach for patients with glioma.

## CONCLUSION

Microsurgery demonstrates a more favorable clinical efficacy for patients with glioma compared with traditional craniotomy. This treatment approach proves to be both safe and effective as it can facilitate better recovery of serum biomarkers such as AVP, OT, and β-EP, exert a minimal impact on inflammatory markers, and effectively improve patients’ cognitive function and ADL.

### Authors’ Contributions:

**YC** and **ZM:** Conceived and designed the study.

**DZ** and **LM:** Collected the data and performed the analysis.

**XS:** Was involved in the writing of the manuscript and is responsible for the integrity of the study.

All authors have read and approved the final manuscript.

## References

[1] Xu S, Tang L, Li X, Fan F, Liu Z (2020). Immunotherapy for glioma:Current management and future application. Cancer Lett.

[2] Erices JI, Bizama C, Niechi I, Uribe D, Rosales A, Fabres K (2023). Glioblastoma Microenvironment and Invasiveness:New Insights and Therapeutic Targets. Int J Mol Sci.

[3] Xiong Z, Xiong Y, Liu H, Li C, Li X (2020). Identification of purity and prognosis-related gene signature by network analysis and survival analysis in brain lower grade glioma. J Cell Mol Med.

[4] Tsitlakidis A, Aifantis EC, Kritis A, Tsingotjidou AS, Cheva A, Selviaridis P (2020). Mechanical properties of human glioma. Neurol Res.

[5] Fujii Y, Ogiwara T, Goto T, Kanaya K, Hara Y, Hanaoka Y (2021). Microscopic Navigation-Guided Fence Post Technique for Maximal Tumor Resection During Glioma Surgery. World Neurosurg.

[6] Schaff LR, Mellinghoff IK (2023). Glioblastoma and Other Primary Brain Malignancies in Adults:A Review. JAMA.

[7] Wang LM, Englander ZK, Miller ML, Bruce JN (2023). Malignant Glioma. Adv Exp Med Biol.

[8] Bai J, Varghese J, Jain R (2020). Adult Glioma WHO Classification Update, Genomics, and Imaging:What the Radiologists Need to Know. Top Magn Reson Imaging.

[9] Jia X, Wang Z, Huang F, Su C, Du W, Jiang H (2021). A comparison of the Mini-Mental State Examination (MMSE) with the Montreal Cognitive Assessment (MoCA) for mild cognitive impairment screening in Chinese middle-aged and older population:a cross-sectional study. BMC Psychiatry.

[10] Tramontano M, Belluscio V, Bergamini E, Allevi G, De Angelis S, Verdecchia G (2022). Vestibular Rehabilitation Improves Gait Quality and Activities of Daily Living in People with Severe Traumatic Brain Injury:A Randomized Clinical Trial. Sensors (Basel).

[11] Singh M, Raghav A, Gautam KA (2022). Role of the circulatory interleukin-6 in the pathogenesis of gliomas:A systematic review. World J Methodol.

[12] Diaz S, Reyns N, Özduman K, Levivier M, Schulder M, Tuleasca C (2023). Microsurgical resection of gliomas of the cingulate gyrus:a systematic review and meta-analysis. Neurosurg Rev.

[13] Patti G, Calandra E, De Bellis A, Gallizia A, Crocco M, Napoli F (2020). Antibodies Against Hypothalamus and Pituitary Gland in Childhood-Onset Brain Tumors and Pituitary Dysfunction. Front Endocrinol (Lausanne).

[14] Sayyahmelli S, Sayyahmelli S, Ozaydin B, Erginoglu U, Keleş A, Sun Z (2022). Safety and efficacy of microsurgery for complex cranial pathologies in the ultra-geriatric population. Clin Neurol Neurosurg.

[15] Xu J, Zhang J, Zhang Z, Gao Z, Qi Y, Qiu W (2021). Hypoxic glioma-derived exosomes promote M2-like macrophage polarization by enhancing autophagy induction. Cell Death Dis.

[16] Bashir F, Qureshi BM, Minhas K, Tabori U, Bouffet E, Hawkins C (2023). Pakistan National Guidelines for Pediatric High-Grade Gliomas. Pak J Med Sci.

[17] Song B, Wang X, Qin L, Hussain S, Liang W (2024). Brain gliomas:Diagnostic and therapeutic issues and the prospects of drug-targeted nano-delivery technology. Pharmacol Res.

[18] Izadpanah A, Moran SL (2020). Pediatric Microsurgery:A Global Overview. Clin Plast Surg.

[19] Förnvik K, Maddahi A, Liljedahl E, Osther K, Salford LG, Redebrandt HN (2021). What is the role of CRP in glioblastoma?. Cancer Treat Res Commun.

[20] Feng Y, Wang J, Tan D, Cheng P, Wu A (2019). Relationship between circulating inflammatory factors and glioma risk and prognosis:A meta-analysis. Cancer Med.

